# Telehealth Scale and Artificial Intelligence Adoption Tiers Across Clinical and Operational Domains in US Hospitals: Cross-Sectional Study

**DOI:** 10.2196/96762

**Published:** 2026-06-12

**Authors:** Lingbo Liu

**Affiliations:** 1 Thrust of Urban Governance and Design, Society Hub, The Hong Kong University of Science and Technology (Guangzhou), No 1 Du Xue Rd, Nansha District, Guangzhou, Guangdong, China, 86 18971571598; 2 Center for Geographic Analysis, Harvard University, Cambridge, MA, United States

**Keywords:** artificial intelligence, telemedicine, telehealth, digital health, hospitals, health information technology, machine learning, explainable artificial intelligence, extreme gradient boosting, XGBoost, Shapley additive explanations, United States

## Abstract

**Background:**

Telehealth expansion and artificial intelligence (AI) adoption are often described as parallel dimensions of health system digital transformation. However, whether telehealth scale is associated with hospital AI adoption and whether this relationship varies across hospital settings remain unclear.

**Objective:**

This study examined the association of telehealth scale with clinical and operational AI adoption tiers in US hospitals and assessed whether these patterns differed by telehealth reporting behavior and geography.

**Methods:**

This cross-sectional study included 6173 US acute care hospitals using linked 2024 American Hospital Association Annual Survey and Information Technology Supplement data and 2023 Healthcare Cost Report Information System data. Telehealth scale was parameterized using log-transformed telehealth volume, a telehealth nonreporting indicator, and a reported-zero telehealth indicator. Clinical and operational AI adoption tiers were derived from hospital-reported AI capability items and classified into 3 tiers. Both outcomes were modeled using multioutput gradient-boosted tree classifiers, and model behavior was interpreted using Shapley additive explanations, partial dependence plots, and stratified analyses by the Core-Based Statistical Area category.

**Results:**

Telehealth volume was the strongest predictor of both clinical and operational AI adoption tiers and had a larger contribution to the clinical AI model. Telehealth nonreporting was common, occurring in 57% (3521/6173) of hospitals, and was concentrated among hospitals in the lowest clinical AI adoption tier, accounting for 91.4% (3145/3441) of hospitals with no reported clinical AI adoption. Higher telehealth volume was associated with a steep increase in predicted clinical AI adoption tiers at lower telehealth volumes, followed by a plateau at higher volumes. At similar telehealth volumes, rural hospitals showed weaker telehealth-attributed contributions to predicted clinical AI adoption tiers than metropolitan hospitals. Supplementary analyses suggested that telehealth reporting status and telehealth intensity reflected related but distinct structural processes.

**Conclusions:**

Telehealth scale was strongly associated with hospital AI adoption tiers, especially clinical AI adoption tiers. These findings suggest that telehealth capacity may serve as a practical hospital-level marker of broader digital readiness for AI adoption, but the cross-sectional design does not establish whether telehealth expansion precedes or causes AI adoption. Hospitals with telehealth nonreporting and rural hospitals may face additional structural barriers that limit the translation of digital capacity into AI maturity. Policies to reduce inequities in hospital AI adoption may therefore need to pair telehealth expansion with implementation support, interoperability capacity, and organizational resources.

## Introduction

Telehealth expansion and artificial intelligence (AI) adoption are 2 prominent dimensions of the ongoing digital transformation in health care [1]. Telehealth use increased rapidly during and after the COVID-19 pandemic and has remained an important channel for care delivery, while AI has been introduced across a growing range of clinical and operational functions in hospitals [2-4]. Although these developments are often discussed separately, both depend on a shared foundation of digital infrastructure, data availability, workflow integration, and organizational capacity [5-7]. Hospitals that are able to deliver care at scale through digital platforms may also be better positioned to implement AI tools that rely on timely, structured, and interoperable information [8,9]. This shared infrastructure perspective is important because hospital digital transformation is shaped not only by organizational and implementation capacity but also by the availability of individual technologies. Adopting and sustaining digital tools requires data infrastructure, workflow integration, governance, implementation planning, and organizational readiness [10]. National studies suggest that advanced digital capabilities remain unevenly distributed across US hospitals, with variation by hospital size, metropolitan status, and local context [11,12]. Telehealth scale may therefore provide an observable hospital-level marker of broader digital readiness.

From a health informatics perspective, AI implementation requires more than access to algorithms. It depends on the ability of health systems to capture digital patient interactions, integrate data across platforms, support technology-enabled workflows, and maintain governance structures that enable safe and sustained use [13-15]. Telehealth may therefore provide a useful systems-level indicator of broader digital readiness. Hospitals with higher telehealth activity may have already invested in patient-facing digital interfaces, staff training, data exchange processes, and operational coordination that also support AI deployment, particularly for clinical applications [16]. By contrast, hospitals with limited telehealth infrastructure may face barriers that also constrain the adoption of AI-enabled tools [17-20].

This question has important equity implications. Prior research suggests that digital capacity is unevenly distributed across hospitals and communities, with rural, safety-net, and socioeconomically disadvantaged settings often facing greater limitations in health information technology infrastructure, broadband access, staffing, and implementation support [21-23]. These constraints may affect not only telehealth access but also the ability to adopt and operationalize AI in routine care. If telehealth scale is associated with hospital AI maturity, then uneven telehealth capacity may signal a broader digital divide in AI readiness [24-27]. Understanding this relationship is therefore relevant not only for innovation strategy but also for equitable health system transformation [28-31].

Despite growing interest in both telehealth and AI, the relationship between telehealth scale and hospital AI maturity has not been well characterized at the national level. In addition, telehealth nonreporting may reflect a structurally different state from explicitly reported-zero telehealth use, but these 2 conditions are often not distinguished analytically. Using data from 6173 US hospitals, this study examined whether telehealth scale was associated with clinical and operational AI adoption tiers, whether telehealth nonreporting and reported-zero telehealth volume showed different patterns, and whether these relationships varied across geographic settings. The study hypothesized that telehealth scale would be positively associated with both domains of AI adoption tiers, with stronger associations for clinical AI, and that rural hospitals would show weaker telehealth-associated AI readiness at comparable telehealth volumes.

## Methods

### Study Design and Data Sources

This retrospective cross-sectional study followed the STROBE (Strengthening the Reporting of Observational Studies in Epidemiology) reporting guideline. The study setting comprised US acute care hospitals represented in the linked 2024 American Hospital Association (AHA) Annual Survey, AHA Information Technology Supplement, and 2023 Healthcare Cost Report Information System (HCRIS) datasets. The hospital, rather than the patient or encounter, served as the unit of analysis. Since this was a secondary analysis of hospital-level administrative and survey data, no participant recruitment was conducted. The 2024 AHA Annual Survey and Information Technology Supplement were linked with hospital utilization and financial data from the 2023 HCRIS to construct a national analytic dataset of US acute care hospitals. Hospitals were matched using facility identifiers and retained if they had valid linkage keys and met acute care eligibility criteria, resulting in a final analytic sample of 6173 hospitals. Financial variables were expressed in 2023 US dollars. Detailed variable definitions and coding rules are provided in Table S1 in Multimedia Appendix 1.

### Exposures, Outcomes, and Covariates

The primary exposure was annual telehealth video-visit volume from the AHA survey. To distinguish reporting behavior from observed telehealth use, telehealth was parameterized using 3 variables: log-transformed telehealth volume, a telehealth nonreporting indicator, and a reported-zero indicator among hospitals that submitted a number of telehealth video visits. This parameterization allowed hospitals with no reported telehealth value to be distinguished from hospitals that explicitly reported zero telehealth volume. Telehealth nonreporting was treated as a distinct analytic state rather than ordinary random missingness because it may reflect limited telehealth capability, limited administrative reporting infrastructure, survey response capacity, reimbursement context, or broader digital disadvantage. Reported-zero telehealth volume was interpreted as active nonuse or absence of telehealth service delivery during the reporting period. The primary outcomes were clinical AI adoption tiers and operational AI adoption tiers, constructed from AHA survey items. The clinical AI domain included AI use for diagnosis, patient care activities, clinical decision support, robotic or assisted surgery, precision medicine or genetic analysis, population health management, medication administration, and other clinical applications. The operational AI domain included AI use for revenue cycle management, supply chain management, staffing and scheduling, patient flow and discharge planning, operational efficiency, and applications of information technology or facilities. For each domain, cleaned item responses were summed to generate a raw score, and hospitals were classified into 3 empirical AI adoption tiers: tier 0 for nonresponse across all domain items or a summed score of 0, tier 1 for active adopters at or below the median score among adopters, and tier 2 for active adopters above that median. Complete nonresponse and zero adoption were combined in tier 0 because both states indicate no reported AI functionality in the available AHA survey items, and the data do not provide sufficient information to reliably distinguish the absence of adoption from the absence of item-level reporting. Therefore, tier 0 was interpreted conservatively as no reported AI adoption rather than as a validated maturity stage or definitive evidence of no AI use. Because no externally validated national hospital AI maturity scale was available in these data, these categories were interpreted as empirical adoption tiers rather than formally validated maturity stages.

Candidate covariates were selected to capture hospital scale, throughput, capital intensity, payer mix, safety-net burden, organizational structure, and geography. The linked datasets did not include direct measures of AI-specific or telehealth-specific investment, such as dedicated digital health budgets, vendor contracts, implementation teams, or specialized technical staffing. Therefore, financial and organizational variables, including total operating expenses, payroll expenses, capital intensity, teaching status, and system affiliation, were used as indirect proxies for organizational resource capacity. These variables included total patient visits, admissions, staffed beds, surgical volume, total operating expenses, payroll, Medicaid and Medicare inpatient days, teaching status, system affiliation, and the Core-Based Statistical Area category. Capital intensity was defined as total operating expenses per staffed bed, and surgical intensity as inpatient surgeries divided by admissions plus 1. Numeric variables were coerced after standardizing missing-value tokens; remaining missing numeric predictors were set to 0 for tree-based modeling. Missing or nonpositive bed counts were replaced with the median of positive values to avoid implausible denominators, and categorical variables were represented using indicator variables.

### Statistical Analysis

Hospital characteristics were summarized overall and by clinical AI adoption tier using medians and IQRs for skewed continuous variables and counts with percentages for categorical variables; differences across tiers were assessed using Kruskal-Wallis and χ^2^ tests. To visualize regional patterns, hospital-level measures were aggregated to the county level, and local spatial autocorrelation for county-level telehealth intensity and clinical AI was assessed using local indicators of spatial association based on county adjacency. The main predictive analysis used multioutput gradient-boosted tree classification to model clinical and operational AI adoption tiers simultaneously from a shared feature set, implemented as parallel multiclass extreme gradient boosting (XGBoost) models with an 80%/20% train-test split stratified on the joint clinical-operational tier label when feasible and otherwise on the clinical AI tier. The XGBoost classifiers used 300 estimators, a learning rate of 0.03, a maximum tree depth of 5, subsampling of 0.8, column subsampling of 0.8, multiclass soft-probability objectives, and log-loss evaluation. Model performance was evaluated on a held-out 20% test set.

To interpret model behavior, SHAP (Shapley additive explanations) values were estimated for the tier 2 outcome, and global feature importance was summarized using mean absolute SHAP values. SHAP values were used to quantify feature contributions to model predictions and should not be interpreted as causal effects, independent risk ratios, or direct policy levers. Signed SHAP contributions for telehealth nonreporting and reported-zero telehealth volume were also calculated to distinguish their associations with high AI adoption. Partial dependence plots were used to assess nonlinear associations for prespecified drivers, including telehealth intensity, telehealth nonreporting, expenses per bed, and total patient visits. Geographic heterogeneity in telehealth-attributed clinical AI readiness was evaluated by stratifying SHAP dependence by the Core-Based Statistical Area category and smoothing these patterns with locally weighted regression.

As sensitivity analyses, 2 additional sets of models were conducted. First, to assess whether the main results were driven by structural telehealth nonreporting, the analytic sample was restricted to hospitals that reported telehealth volume, and the clinical and operational AI tier models were reestimated after removing the telehealth nonreporting indicator. In these reporter-only models, telehealth scale was represented by log-transformed reported telehealth volume and an indicator for reported-zero telehealth volume. Second, to evaluate whether the findings depended on the distribution-based AI tier definitions, the 3-level clinical and operational AI adoption tier outcomes were replaced with the corresponding continuous raw AI scores, and XGBoost regression models were estimated using the same predictor set as the primary analysis. Two-sided *P*<.001 was considered statistically significant for descriptive comparisons. Analyses were conducted in Python (Python Software Foundation). Detailed model specifications and full performance metrics are provided in Multimedia Appendix 1.

### Ethical Considerations

This study used deidentified, institution-level secondary administrative and survey data, with hospitals as the unit of analysis. It did not involve patient-level records, protected health information, or interaction with human participants. The present analysis was not submitted for institutional review board review because it did not constitute human participants’ research under relevant institutional policy [32] and applicable regulations for secondary analyses of deidentified institutional data. No informed consent was required. The author had licensed permission to use the AHA data [33], and HCRIS data were used in accordance with its access conditions. No ethics application number was assigned.

## Results

### Sample Characteristics by the Clinical AI Maturity Tier

The analytic cohort included 6173 US hospitals. When hospitals were stratified by clinical AI adoption tiers, 3441 (55.7%) were classified as tier 0, 1544 (25%) as tier 1, and 1188 (19.2%) as tier 2 (Table 1). Clinical AI scores increased from a median of 0 (IQR 0‐0) in tier 0 to 22 (IQR 19‐25) in tier 2, while operational AI scores increased from 0 (IQR 0‐0) to 14 (IQR 11‐17; *P*<.001 for both). Telehealth volume increased in parallel, from a median of 0.00 thousand visits (IQR 0.00‐0.74) in tier 0 to 2.40 (IQR 0.21‐20.93) in tier 2 (*P*<.001). Tier 2 hospitals also had greater patient throughput and capital intensity than tier 0 hospitals, with median total visits increasing from 34.17 thousand (IQR 9.14‐107.00) to 160.37 (IQR 75.13‐331.87) and median expenses per bed increasing from $1.08 million (IQR $0.49-$1.90 million) to $1.82 million (IQR $1.33-$2.54 million; *P*<.001 for both). Geographic and institutional characteristics were similarly stratified: tier 2 hospitals were predominantly urban (927/1188, 78%), while rural hospitals were underrepresented in tier 2 (102/1188, 8.6%) and more common in tier 1 (395/1544, 25.6%). Teaching hospitals were also more concentrated in tier 2 than tier 0 (157/1188, 13.2% vs 72/3441, 2.1%; *P*<.001).

**Table 1.** Hospital characteristics by the clinical artificial intelligence (AI) adoption tier^a^.

Variable	Overall (N=6173)	Tier 0 (no reported adoption; n=3441)	Tier 1 (low; n=1544)	Tier 2 (high; n=1188)	*P* value
Clinical AI score (0‐40), median (IQR)	0 (0-14)	0 (0-0)	11 (8-14)	22 (19-25)	<.001
Operational AI score (0‐30), median (IQR)	0 (0-10)	0 (0-0)	8 (6-10)	14 (11-17)	<.001
Telehealth volume (thousands of visits), median (IQR)	0.41 (0.00-5.44)	0.00 (0.00-0.74)	0.09 (0.00-1.94)	2.40 (0.21-20.93)	<.001
Total patient visits (thousands), median (IQR)	57.88 (15.47-162.81)	34.17 (9.14-107.00)	60.95 (18.51-157.58)	160.37 (75.13-331.87)	<.001
Expenses per bed ($M/bed), median (IQR)	1.32 (0.64-2.13)	1.08 (0.49-1.90)	1.31 (0.65-2.12)	1.82 (1.33-2.54)	<.001
Total operating expenses ($M), median (IQR)	75.84 (30.76-273.50)	54.10 (27.67-169.66)	65.86 (26.24-232.15)	275.28 (93.93-654.33)	<.001
Surgical intensity ratio, median (IQR)	0.76 (0.12-1.59)	0.68 (0.00-1.82)	0.80 (0.06-1.57)	0.83 (0.57-1.25)	<.001
Telehealth reporting status, n (%)					<.001
Telehealth volume not reported	3521 (57.0)	3145 (91.4)	248 (16.1)	128 (10.8)	
Reported-zero telehealth volume (among reporters), n (%)					<.001
True zero	830 (31.3)	180 (60.8)	515 (39.7)	135 (12.7)	
Location, n (%)					<.001
Urban	4223 (68.4)	2373 (69.0)	923 (59.8)	927 (78.0)	
Micro	852 (13.8)	467 (13.6)	226 (14.6)	159 (13.4)	
Rural	1098 (17.8)	601 (17.5)	395 (25.6)	102 (8.6)	
Teaching status, n (%)					<.001
Teaching	278 (4.5)	72 (2.1)	49 (3.2)	157 (13.2)	
Nonteaching	5895 (95.5)	3369 (97.9)	1495 (96.8)	1031 (86.8)	

a Values for categorical variables are presented as n/N (%), where percentages are column percentages unless otherwise specified. Reported-zero telehealth volume is calculated among hospitals that reported a telehealth volume value.

Telehealth reporting status also varied sharply across tiers. Overall, 3521 out of 6173 (57%) hospitals did not report telehealth volume. Nonreporting was concentrated in tier 0 (3145/3441, 91.4%) and was uncommon in tier 2 (128/1188, 10.8%; *P*<.001). Among hospitals that reported telehealth volume, reported-zero telehealth use was most frequent in tier 0 (180/296, 60.8%) and least frequent in tier 2 (135/1060, 12.7%), suggesting that telehealth nonreporting and reported-zero use represent related but distinct states of digital capability.

### Spatial Concordance of Telehealth Capacity and Clinical AI

**Figure 1.**
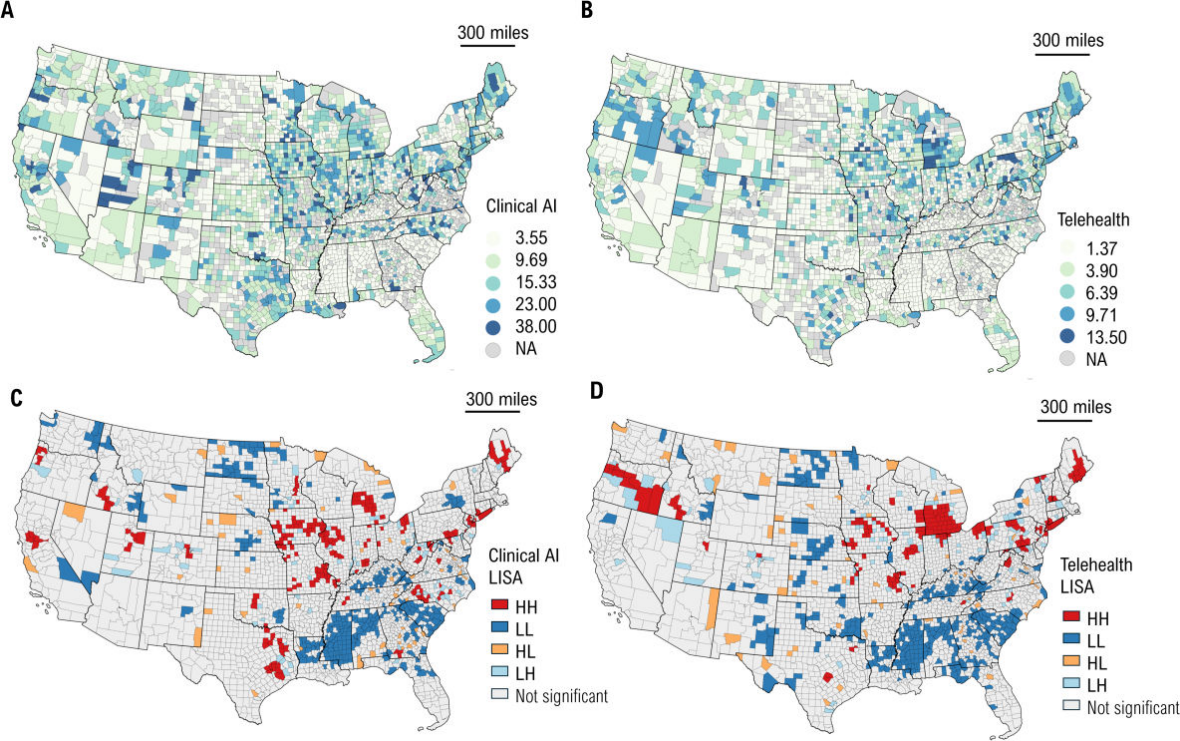
Spatial concordance and clustering of telehealth utilization and clinical artificial intelligence (AI) maturity across continental US counties. (B) County-level mean logarithmic telehealth volume. (A) County-level mean clinical AI score. The 2 raw-value maps show strong spatial concordance, with higher telehealth activity and higher clinical AI colocated in major metropolitan corridors. (C) LISA classification for clinical AI, showing a similar hot spot and cold spot structure. (D) Local indicators of spatial association (LISA) for log telehealth volume, expressed as local Moran I *z*-scores and classified into high-high (HH), low-low (LL), high-low (HL), low-high (LH), and not significant clusters.

County-level maps showed substantial spatial concordance between log-transformed telehealth volume and clinical AI adoption tiers (Figure 1). Counties with higher telehealth activity tended to colocate with higher clinical AI scores, whereas counties with lower telehealth activity tended to colocate with lower clinical AI scores. Local indicators of spatial association further indicated that these patterns were spatially clustered rather than diffuse. High-high clusters for telehealth volume were concentrated along major metropolitan and coastal corridors, including the Northeast and parts of the West Coast, and clinical AI showed a similar clustering pattern. Low-low clusters extended across parts of the South, Appalachia, and the Great Plains, consistent with regionally organized deficits in digital capacity.

### Telehealth Scale and AI Adoption Tiers in Predictive Models

**Figure 2.**
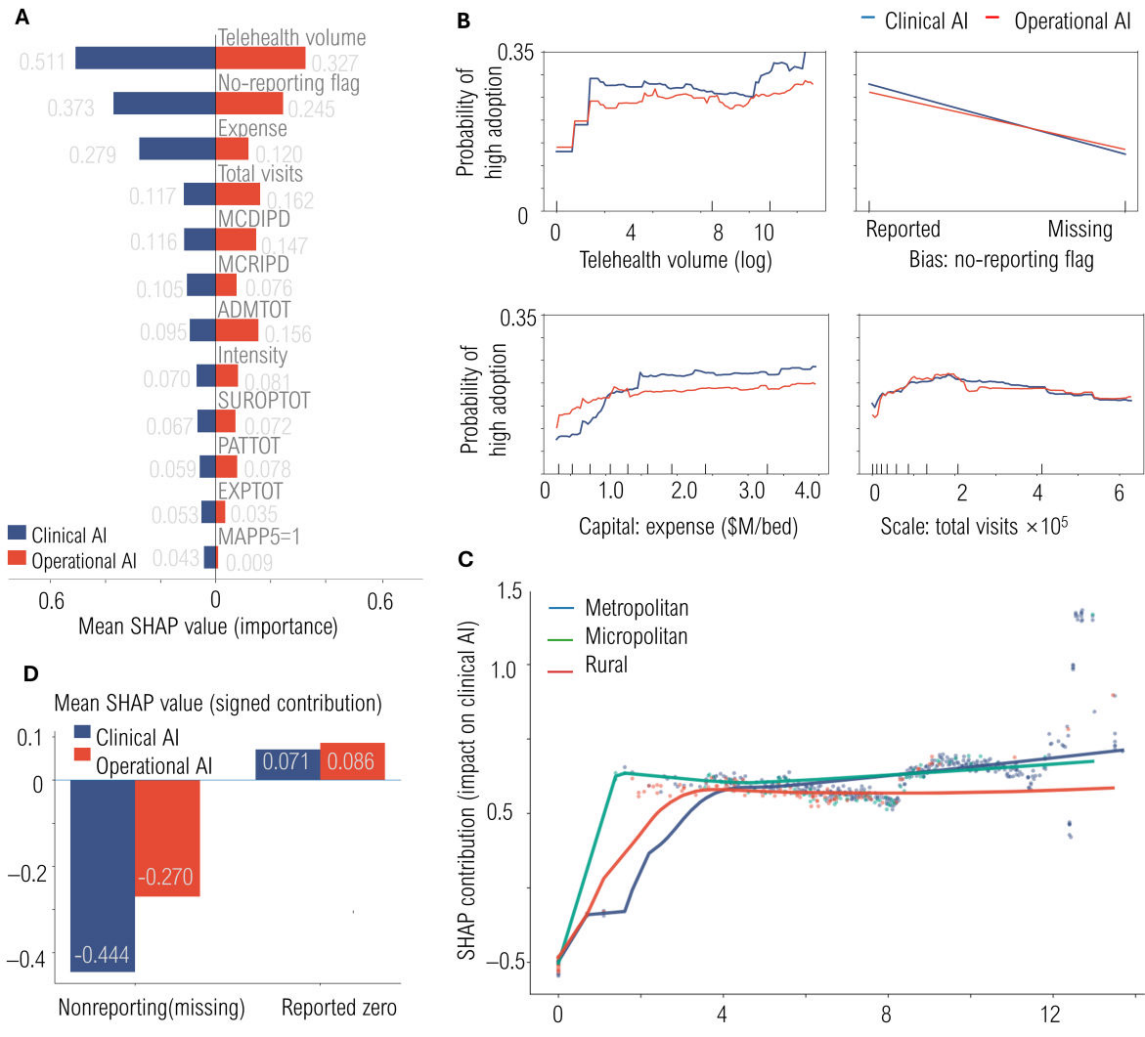
Telehealth scale as a model-based marker of hospital artificial intelligence (AI) adoption tiers. (A) Global feature importance from Shapley additive explanations (SHAP), summarized as mean absolute SHAP values for the probability of high adoption, defined as tier 2, comparing clinical AI and operational AI models. Telehealth volume, log-transformed, was the top predictor in both domains and had a larger model contribution in the clinical AI model. (B) Partial dependence plots showing model-predicted probability of tier 2 adoption across telehealth volume, telehealth nonreporting, defined as missing VIDVZ, capital intensity, defined as expenses per bed, and total patient visits; rug marks indicate the observed feature distribution. (C) Geography-stratified SHAP dependence of telehealth volume for the clinical AI model, with LOESS-smoothed trends and point estimates, showing lower telehealth-attributed contributions in rural hospitals than in metropolitan hospitals at comparable volumes. (D) Mean signed SHAP contributions for telehealth nonreporting and reported-zero telehealth volume, defined as VIDVZ=0 among reporting hospitals, shown for clinical and operational AI models; negative values indicate reduced probability of tier 2 adoption. ADMTOT: total admissions; EXPTOT: total operating expenses; Intensity: surgical intensity ratio; LOESS: locally estimated scatterplot smoothing; MAPP5=1: hospital affiliated with a larger health system; MCDIPD: Medicaid inpatient days; MCRIPD: Medicare inpatient days; PATTOT: total patient visits; SUROPTOT: total surgical operations; VIDVZ: number of telehealth video visits.

In the multioutput XGBoost models predicting tier 2 adoption, telehealth volume was the strongest predictor of both clinical and operational AI adoption tiers (Figure 2A). The mean absolute SHAP value for telehealth volume was 0.511 in the clinical AI model and 0.327 in the operational AI model, indicating a stronger association with clinical AI than operational AI. Telehealth nonreporting also ranked highly in both models (mean absolute SHAP=0.373 for clinical AI and 0.245 for operational AI). Capital intensity was more predictive for clinical AI than operational AI (mean absolute SHAP value=0.279 vs 0.120), whereas throughput-related variables contributed relatively more to operational AI. Partial dependence plots showed a nonlinear association between telehealth volume and predicted clinical AI adoption tiers (Figure 2B). The predicted probability of tier 2 clinical AI adoption increased steeply during early telehealth scale-up and then plateaued at higher telehealth volumes. By contrast, telehealth nonreporting was associated with a downward shift in predicted adoption probability across the feature range. Capital intensity showed a saturating association, while total visits had a weaker and less specific association with clinical AI readiness. On the held-out test set, the clinical AI tier model achieved an overall accuracy of 0.777 and a Cohen κ of 0.618, while the operational AI tier model achieved an overall accuracy of 0.776 and a Cohen κ of 0.607. Class-specific performance metrics are reported in Multimedia Appendix 1.

### Geographic Heterogeneity, Reporting Mechanisms, and Robustness Analyses

Geographic stratification of SHAP dependence suggested that, at comparable telehealth volumes, rural hospitals consistently had lower telehealth-associated contributions to predicted clinical AI adoption tiers than metropolitan hospitals (Figure 2C). In addition, signed SHAP values suggested that telehealth nonreporting was associated with a negative contribution to the probability of tier 2 adoption, particularly for clinical AI, whereas reported-zero telehealth volume showed a smaller and directionally distinct pattern (Figure 2D).

**Figure 3.**
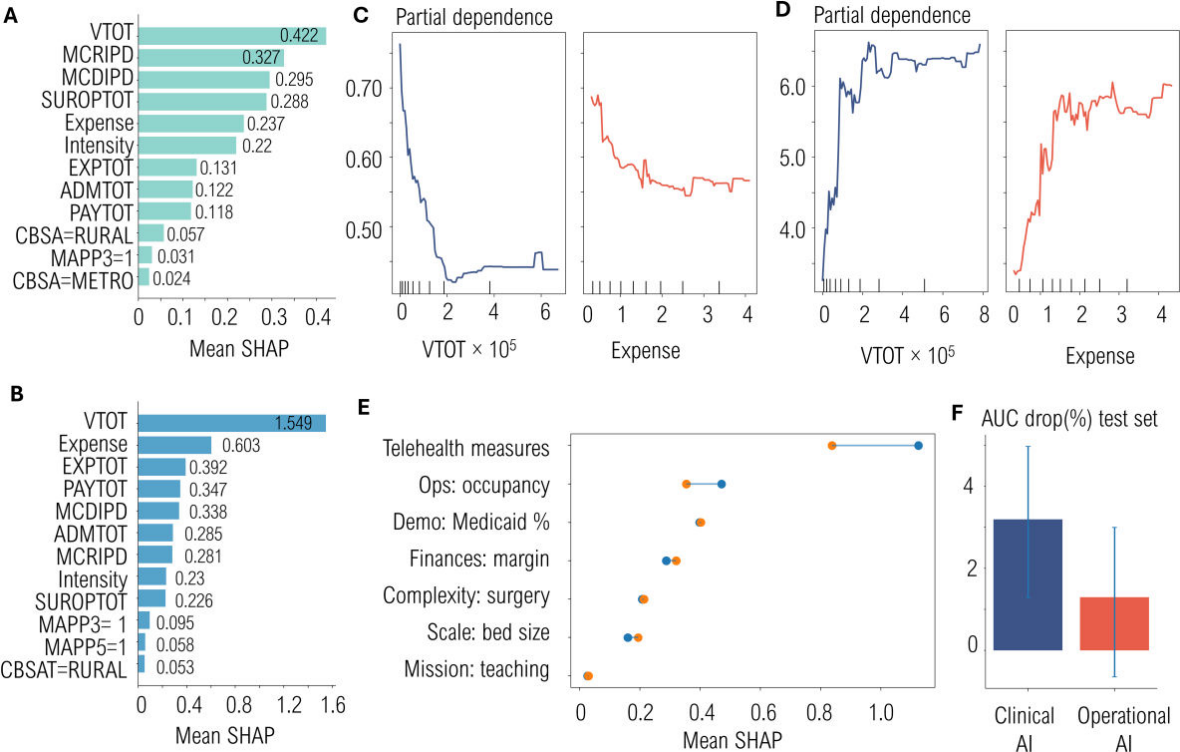
Decomposing telehealth reporting and intensity and testing robustness. (A) Global feature importance, summarized as mean absolute Shapley additive explanations (SHAP) values, for a reporting model predicting telehealth nonreporting, defined as missing annual telehealth video-visit volume (VIDVZ), using hospital scale, utilization, and financial covariates. (B) Global feature importance, summarized as mean absolute SHAP values, for an intensity model predicting telehealth volume among reporting hospitals, with the outcome defined as log (1+VIDVZ). (C) Partial dependence plots (PDPs) for the reporting model showing the modeled probability of telehealth nonreporting as a function of total visits (VTOT) and capital intensity, defined as expenses per bed. (D) PDPs for the intensity model, restricted to reporting hospitals, showing predicted telehealth volume on the log scale as a function of VTOT and expenses per bed; rug marks indicate the distribution of observations. (E) Competing-drivers analysis of high artificial intelligence (AI) adoption: mean absolute SHAP values, computed on the held-out test set, comparing telehealth measures with alternative explanatory factors, including financial pressure, defined as operating margin; organizational scale, defined as bed size; mission, defined as teaching status; complexity, defined as surgery intensity; payer mix, defined as Medicaid ratio; and operational pressure, defined as occupancy proxy, shown separately for clinical and operational AI high adoption. (F) Ablation test quantifying the percentage drop in the test set area under the receiver operating characteristic curve (AUC) after removing the telehealth feature from the competing-drivers models for clinical and operational AI; larger drops indicate greater dependence of model discrimination on the telehealth-related predictive signal. ADMTOT: total admissions; CBSA: Core-Based Statistical Area; CBSA=METRO: hospital located in a metropolitan CBSA; CBSA=RURAL: hospital located outside metropolitan or micropolitan CBSA; EXPTOT: total operating expenses; MAPP3=1: participating site recognized for one or more programs accredited by Accreditation Council for Graduate Medical Education; EXPTOT: total operating expenses; Intensity: surgical intensity ratio; MAPP5=1: hospital affiliated with a larger health system; MCDIPD: Medicaid inpatient days; MCRIPD: Medicare inpatient days; PAYTOT: total payroll expenses; SUROPTOT: total surgical operations; VTOT: total patient visits.

Supplementary analyses further suggested that telehealth reporting status and telehealth intensity reflected distinct structural processes. A separate gradient-boosted model predicted telehealth nonreporting with good discrimination (test area under the receiver operating characteristic curve [AUC]=0.814), and among reporting hospitals, a telehealth intensity model explained substantial variation in log-transformed telehealth volume (test *R*²=0.41; Tables S3 and S4 in Multimedia Appendix 1). In competing-driver analyses, telehealth remained the largest contributor to high adoption in both AI domains, and in ablation analyses, removing telehealth reduced test-set discrimination, with a larger decline for clinical AI than for operational AI (Figure 3; Tables S5 and S6 in Multimedia Appendix 1).

### Sensitivity Analyses

Sensitivity analyses further supported the robustness of the main findings while highlighting the structural importance of telehealth nonreporting. In reporter-only models restricted to hospitals with reported telehealth volume (n=2652), log-transformed telehealth volume remained the strongest predictor of high clinical and operational AI adoption. The reporter-only clinical model achieved an accuracy of 0.653, a Cohen κ of 0.374, and a tier 2 AUC of 0.821. The reporter-only operational model achieved an accuracy of 0.633, a Cohen κ of 0.329, and a tier 2 AUC of 0.775. In both models, log-transformed telehealth volume ranked first in SHAP importance for tier 2 adoption, with mean absolute SHAP values of 0.274 for clinical AI and 0.198 for operational AI.

Continuous-score sensitivity analyses showed similar patterns when the clinical and operational AI raw scores were modeled directly rather than categorized into 3 tiers. The clinical AI score model achieved an *R*² of 0.590, a root mean square error of 6.085, and a mean absolute error of 3.851, while the operational AI score model achieved an *R*² of 0.545, a root mean square error of 4.363, and a mean absolute error of 2.763. Telehealth nonreporting was the strongest predictor of both clinical and operational AI raw scores, and log-transformed telehealth volume was the second strongest predictor in both models. These findings suggest that the main results were not driven solely by telehealth nonreporting or by the median-based construction of AI adoption tiers.

## Discussion

### Principal Findings

In this cross-sectional study of 6173 US hospitals, telehealth volume was strongly associated with hospital AI adoption tiers and was more strongly associated with clinical AI than with operational AI. Across descriptive, spatial, and model-based analyses, 3 findings were notable: telehealth volume showed the largest model contribution to high AI adoption in both domains, the association with clinical AI adoption tiers appeared nonlinear, and telehealth nonreporting and rural location were both associated with lower predicted clinical AI adoption tiers. Together, these findings suggest that telehealth capacity may serve as a model-based marker of broader digital infrastructure and organizational readiness relevant to clinical AI adoption, but the cross-sectional design does not establish whether telehealth expansion precedes or causes AI adoption.

These results also help clarify the relationship between telehealth and AI, which are often discussed in separate policy and implementation literature. Telehealth is commonly framed as an access strategy [5-7], whereas AI is more often framed as an innovation, quality, or efficiency strategy [22]. In these data, however, telehealth volume was more strongly associated with clinical AI maturity than with operational AI maturity. The stronger association with clinical AI than operational AI may reflect the closer alignment between telehealth and patient-facing clinical workflows. Telehealth delivery requires digital patient intake, scheduling, documentation, billing, clinician engagement, and electronic health record integration, which may also support clinical AI applications that depend on structured patient data and clinical decision-support workflows [9,10]. By contrast, operational AI applications may rely more heavily on back-office administrative, staffing, supply-chain, and enterprise resource planning systems. This distinction suggests that clinical and operational AI adoption may be shaped by overlapping but partly different organizational capabilities. This pattern suggests that telehealth may reflect a broader form of digital capability, including routine data capture, platform integration, workflow standardization, and clinician engagement with technology-enabled care, all of which are relevant to equitable digital and AI-enabled care [24]. The weaker association with operational AI is also informative, suggesting that hospital AI adoption may not follow a single pathway and that clinical and operational AI may depend on partly different implementation conditions [9,28].

An additional finding was that telehealth nonreporting was common and concentrated among hospitals in the lowest clinical AI adoption tier. More than half of the hospitals did not report telehealth volume, and additional analyses suggested that nonreporting was itself predictable from hospital structural characteristics and carried a strongly negative contribution to the probability of high AI adoption. This distinction matters because hospitals with missing telehealth data may be systematically different from hospitals with reported-zero telehealth volume. Treating these states as equivalent, or excluding hospitals with missing telehealth values altogether, may therefore obscure an important dimension of the hospital digital divide [18,19]. In this context, telehealth nonreporting should be understood less as a neutral data artifact than as a potential signal of broader digital disadvantage [21].

The geographic findings further highlight the equity implications of these results. County-level clustering showed strong spatial concordance between telehealth intensity and clinical AI maturity, with persistent low-low clusters across parts of the South, Appalachia, and the Great Plains. In addition, at comparable telehealth volumes, rural hospitals showed lower telehealth-associated contributions to predicted clinical AI maturity than metropolitan hospitals. This pattern suggests that telehealth expansion alone may not translate into equivalent AI readiness across settings. Prior work has similarly shown that telemedicine expansion may have limited effectiveness in places with poor internet access, low digital inclusion, and existing health care access constraints, and that hospitals in more socioeconomically deprived service areas are less likely to adopt telehealth and related health information technologies [31]. However, this rural-metro difference should be interpreted as an observed pattern rather than the direct evidence of specific mechanisms. Rural hospitals are heterogeneous, and future work should examine whether this relationship varies by regional context, community deprivation, broadband availability, workforce capacity, and hospital financial resources [8,30].

### Policy and Implementation Implications

These findings have several policy implications. First, policies that support telehealth infrastructure and policies that support AI adoption may be more effective if they are designed together rather than as separate initiatives, because broader digital inclusion and health information capacity shape equitable access to digital care [18]. Second, the nonlinear pattern observed here suggests that the association between telehealth volume and clinical AI maturity may be greater at lower levels of telehealth use than at higher levels. Third, telehealth expansion alone may be insufficient to narrow disparities in AI maturity. Hospitals serving rural and structurally disadvantaged communities may require additional implementation support, including interoperability capacity, workflow redesign, technical staffing, governance, and vendor integration [21]. Overall, efforts to reduce digital inequities in AI adoption may require strengthening multiple components of hospital digital capacity rather than focusing on a single technology alone.

### Limitations

This study has several limitations. First, the cross-sectional design does not establish temporal ordering or causality between telehealth scale and AI adoption. Second, telehealth nonreporting was common and likely structural. Although nonreporting was modeled separately and reporter-only and continuous-score sensitivity analyses were conducted, these strategies cannot fully resolve nonrandom missingness or endogeneity. Third, the AI adoption tiers were empirical measures derived from AHA survey items and distribution-based thresholds rather than externally validated AI maturity stages. Although continuous-score sensitivity analyses using the raw clinical and operational AI scores produced similar telehealth-related patterns, future work should validate hospital AI maturity measures using external implementation benchmarks, governance indicators, workflow integration measures, and longitudinal adoption trajectories. Fourth, the available data did not include the direct measures of AI-specific or telehealth-specific investment, such as dedicated digital health budgets, vendor contracts, implementation teams, specialized technical staffing, or governance capacity. Therefore, financial and organizational variables, including operating expenses, payroll expenses, capital intensity, teaching status, and system affiliation, were used as indirect proxies for organizational resource capacity. Fifth, the analysis did not directly model state-level telehealth regulations, reimbursement policies, broadband policy environments, or regional market conditions, which may influence both telehealth reporting and AI adoption.

### Conclusions

In this national cross-sectional study of US hospitals, telehealth scale was strongly associated with hospital AI adoption tiers, with a particularly strong relationship for clinical AI. Telehealth nonreporting was common and concentrated among hospitals with the lowest clinical AI adoption tiers, and rural hospitals showed weaker telehealth-associated clinical AI patterns than metropolitan hospitals at similar telehealth volumes. These findings suggest that telehealth capacity may serve as a practical hospital-level marker of broader digital readiness for AI adoption, while telehealth nonreporting may identify hospitals at risk of being left behind in digital transformation. From a digital health and health informatics perspective, the results indicate that equitable AI adoption may depend not only on access to AI tools themselves but also on the organizational and infrastructural conditions that support digital care delivery. Efforts to reduce disparities in hospital AI adoption tiers may therefore require combining telehealth expansion with targeted support for interoperability, technical staffing, workflow redesign, governance, and implementation capacity, especially in rural and structurally disadvantaged settings.

## Supplementary material

10.2196/96762 Multimedia Appendix 1 The analysis of telehealth scale and clinical and operational artificial intelligence adoption tiers in US hospitals.

## Abbreviations

AHA: American Hospital Association
AUC: area under the receiver operating characteristic curve
HCRIS: Healthcare Cost Report Information System
SHAP: Shapley additive explanations
STROBE: Strengthening the Reporting of Observational Studies in Epidemiology
XGBoost: extreme gradient boosting
