# Supplementary material for: Telehealth Scale and Artificial Intelligence Adoption Tiers Across Clinical and Operational Domains in US Hospitals: Cross-Sectional Study
Source: J Med Internet Res. 2026 Jun 12;28:e96762. doi: 10.2196/96762 (PMC13263035; doi:10.2196/96762)
Supplement: Multimedia Appendix 1 [file jmir-v28-e96762-s001.docx]

**Association Between Telehealth Volume and Clinical and Operational Artificial Intelligence Adoption Tiers in US Hospitals**

Supplementary Methods, Figure, and Tables

# Methods

## Data preprocessing and feature engineering

Numeric variables were coerced after normalization of common missing-string tokens (eg, "N/A," "NA," and "Not applicable") to standard missing values. Remaining missing numeric predictors were set to 0 for tree-based modeling. Bed denominators were restricted to bed-count fields (BDTOT or BDTOT_D), and missing or nonpositive bed counts were replaced with the median of positive values to avoid implausible denominators. Capital intensity was defined as total operating expenses divided by staffed beds and expressed in millions of 2023 US dollars per bed. Surgical intensity was defined as SUROPTOT divided by ADMTOT plus 1. Categorical predictors, including CBSA category and selected American Hospital Association service-line indicators, were encoded using indicator variables.

## County aggregation and spatial clustering

For geographic visualization, hospital-level telehealth intensity and clinical AI scores were aggregated to the county level. Local spatial autocorrelation was assessed using Local Indicators of Spatial Association based on county adjacency. County maps therefore summarize regional clustering patterns rather than hospital-specific effects and were used to characterize concordance between telehealth capacity and clinical AI maturity across place.

## Multioutput model specification

The primary predictive analysis used MultiOutputClassifier with XGBClassifier to fit 2 parallel multiclass gradient-boosted tree models for clinical AI maturity and operational AI maturity from a shared feature set. Hyperparameters were n_estimators = 300, learning_rate = 0.03, max_depth = 5, subsample = 0.8, colsample_bytree = 0.8, objective = "multi:softprob", num_class = 3, eval_metric = "mlogloss", n_jobs = -1, and random_state = 42. Model performance was evaluated on a held-out 20% test set. Train-test splitting was stratified on the combined clinical-operational tier label when feasible and otherwise on clinical AI tier.

## Model interpretation and supplementary analyses

Model interpretation focused on SHapley Additive exPlanations values for the tier 2 outcome. Global feature importance was summarized as the mean absolute SHAP value. To distinguish the contribution of telehealth reporting behavior from reported zero telehealth volume, signed SHAP summaries were calculated separately for hospitals with Telehealth_Missing = 1 and for hospitals with Telehealth_Zero = 1; negative values indicate reduced model-predicted probability of tier 2 adoption. Partial dependence plots were generated for prespecified drivers, including telehealth intensity, telehealth nonreporting, expenses per bed, and total visits. Geographic modification was evaluated by stratifying telehealth-related SHAP dependence by Core-Based Statistical Area category and smoothing the patterns with locally weighted regression. Supplementary analyses additionally modeled telehealth nonreporting and telehealth intensity separately and included competing-driver and telehealth-ablation analyses. All analyses were conducted in Python.

# Figure S1. Partial dependence plots for prespecified drivers in the XGBoost models

Plots display the modeled functional form for key predictors used in the tier 2 adoption analyses, including telehealth intensity, telehealth nonreporting, expenses per bed, and total visits. Panels are provided to support the nonlinear patterns summarized in the main text.


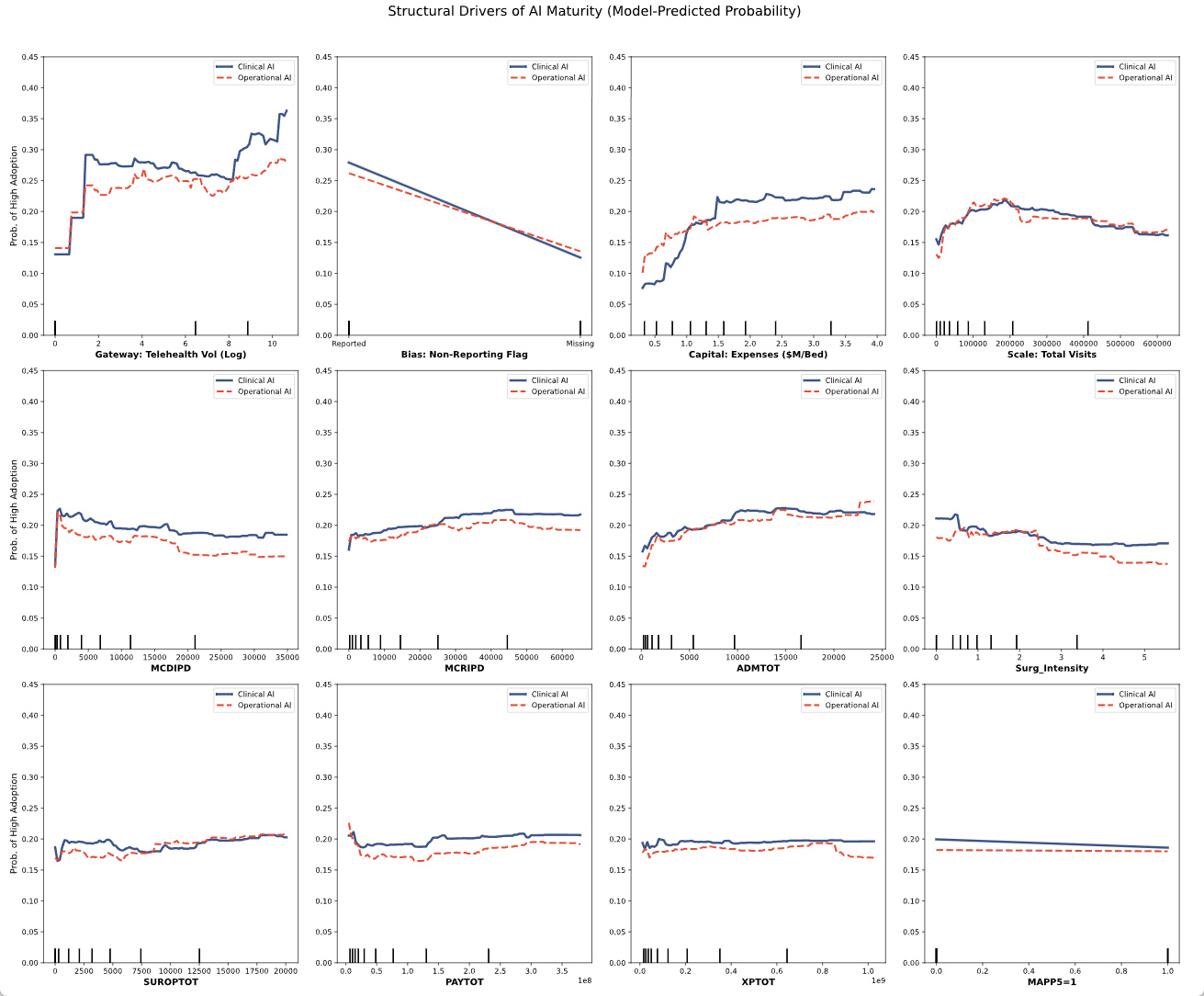


# Table S1. Variable definitions and operationalization

## A. Outcome variables

| **Variable Category** | **Variable Name** | **Definition / Operationalization** |
| --- | --- | --- |
| Clinical AI Maturity | Clin_Tier | Ordinal Target (0, 1, 2). Based on the sum of 8 clinical AI functionalities (see below). Hospitals are classified as: Tier 0: No reported AI adoption, defined as complete domain-item nonresponse or a summed score of 0. Tier 1 (Low): Score ≤ Median of active adopters. Tier 2 (High): Score > Median of active adopters. |
| (Clinical Ingredients) | DIAGAI | AI for Clinical Diagnosis |
| (Clinical Ingredients) | PAPCAI | AI for Patient Care Activities |
| (Clinical Ingredients) | CLINAI | Clinical Decision Support (CDS) |
| (Clinical Ingredients) | SURGAI | AI for Robotic/Assisted Surgery |
| (Clinical Ingredients) | PCOEAI | Precision Medicine / Genetic Analysis |
| (Clinical Ingredients) | POPHAI | Population Health Management |
| (Clinical Ingredients) | PDMRAI | AI for Medication Administration |
| (Clinical Ingredients) | CLNOAI | Other Clinical AI Applications |
| Operational AI Maturity | Oper_Tier | Ordinal Target (0, 1, 2). Based on the sum of 6 operational AI functionalities (see below). Tier logic is identical to Clinical AI. |
| (Operational Ingredients) | AIREVC | AI for Revenue Cycle Management |
| (Operational Ingredients) | AISCOP | AI for Supply Chain Management |
| (Operational Ingredients) | AISSWM | AI for Staffing & Scheduling |
| (Operational Ingredients) | AIPATD | Patient Flow & Discharge Planning |
| (Operational Ingredients) | AIOPEF | AI for Operational Efficiency |
| (Operational Ingredients) | AITIOT | AI for IT & Facilities (IoT) |

## B. Predictor variables

| **Category** | **Variable Name** | **Full Description & Unit** | **Role in Model** |
| --- | --- | --- | --- |
| Telehealth (Primary Predictor) | LOG_VIDVZ | Log Telehealth Volume. Calculated as ln(VideoVisits+1). Missing values are imputed as 0 (controlled by flags). | Telehealth intensity measure: Captures the scale of digitally mediated patient encounters. |
| Telehealth (Primary Predictor) | NA_TH (Telehealth_Is_Missing) | Non-Reporting Flag (Binary). 1 = Hospital did not report telehealth volume; 0 = Reported. | Reporting-status measure: Captures the absence of a reported telehealth volume value. |
| Telehealth (Primary Predictor) | ZERO_TH (Telehealth_Is_Zero) | Zero-Volume Flag (Binary). 1 = Hospital reported explicitly "0" visits; 0 = Reported >0 or Missing. | Capability Control: Distinguishes active non-users from non-reporters. |
| Capital & Financial | EXPOP_BED (Exp_per_Bed_M) | Capital Intensity ($ Millions / Bed).Total Operating Expenses divided by Total Beds. | Capital Proxy: Measures financial liquidity and resource density per unit of capacity. |
| Capital & Financial | EXPTOT | Total Operating Expenses. Total annual costs incurred by the hospital. | Financial Scale. |
| Capital & Financial | PAYTOT | Total Payroll Expenses. Total salary and wage costs. | Labor Cost Structure. |
| Scale & Operations | VTOT | Total Patient Visits. Sum of inpatient and outpatient visits. | Physical Scale: Main driver for Operational AI. |
| Scale & Operations | ADMTOT | Total Admissions. Number of patients admitted to inpatient care. | Throughput volume. |
| Scale & Operations | BDTOT | Total Bed Count. Number of staffed beds. | Physical Capacity. |
| Scale & Operations | SUROPTOT | Inpatient Surgeries. Total surgical operations performed. | Operational Complexity. |
| Scale & Operations | Intensity | Surgical Intensity. Ratio of SUROPTOT / ADMTOT. | Case-mix Complexity. |
| Payer Mix (SES) | MCDIPD | Medicaid Discharge Days. Total inpatient days for Medicaid patients. | Poverty Proxy: Indicates service to low-income populations (Digital Equity). |
| Payer Mix (SES) | MCRIPD | Medicare Discharge Days. Total inpatient days for Medicare patients. | Age/Federal Payer dependence. |
| Structural & Geographic | CBSATYPE | Urban/Rural Status. Categorical: Metro (Urban), Micro, Rural. | Efficiency Gap: Captures geographic barriers to tech activation. |
| Structural & Geographic | MAPP8 | Teaching Status. Categorical: Major, Minor, or Non-teaching. | Academic Affiliation / Research capability. |
| Structural & Geographic | MAPP5 | System Affiliation. Categorical: Member of a health system vs. Independent. | Network Network effects and centralized IT support. |

*Notes. Telehealth intensity was defined as log(1 + VIDVZ). Financial variables were expressed in 2023 US dollars. Categorical variables were represented with indicator variables before modeling. Missing numeric predictors were set to 0, with companion variables used where appropriate to distinguish missingness from observed zero values.*

# Table S2. Model evaluation metrics for the multioutput XGBoost models

| **Outcome (overall accuracy/Cohen kappa)** | **Split** | **Class** | **TP** | **FP** | **TN** | **FN** | **Recall** | **Precision** | **Sensitivity** | **Specificity** | **F1** |
| --- | --- | --- | --- | --- | --- | --- | --- | --- | --- | --- | --- |
| Clinical AI (0.865/0.768) | Train | 0 | 2593 | 283 | 1902 | 160 | 0.942 | 0.902 | 0.942 | 0.87 | 0.921 |
| Clinical AI (0.865/0.768) | Train | 1 | 947 | 232 | 3471 | 288 | 0.767 | 0.803 | 0.767 | 0.937 | 0.785 |
| Clinical AI (0.865/0.768) | Train | 2 | 733 | 150 | 3838 | 217 | 0.772 | 0.83 | 0.772 | 0.962 | 0.8 |
| Operational AI (0.863/0.759) | Train | 0 | 2653 | 278 | 1845 | 162 | 0.942 | 0.905 | 0.942 | 0.869 | 0.923 |
| Operational AI (0.863/0.759) | Train | 1 | 1004 | 275 | 3442 | 217 | 0.822 | 0.785 | 0.822 | 0.926 | 0.803 |
| Operational AI (0.863/0.759) | Train | 2 | 603 | 125 | 3911 | 299 | 0.669 | 0.828 | 0.669 | 0.969 | 0.74 |
| Clinical AI (0.777/0.618) | Test | 0 | 633 | 79 | 468 | 55 | 0.92 | 0.889 | 0.92 | 0.856 | 0.904 |
| Clinical AI (0.777/0.618) | Test | 1 | 201 | 122 | 804 | 108 | 0.65 | 0.622 | 0.65 | 0.868 | 0.636 |
| Clinical AI (0.777/0.618) | Test | 2 | 126 | 74 | 923 | 112 | 0.529 | 0.63 | 0.529 | 0.926 | 0.575 |
| Operational AI (0.776/0.607) | Test | 0 | 644 | 73 | 453 | 65 | 0.908 | 0.898 | 0.908 | 0.861 | 0.903 |
| Operational AI (0.776/0.607) | Test | 1 | 219 | 121 | 783 | 112 | 0.662 | 0.644 | 0.662 | 0.866 | 0.653 |
| Operational AI (0.776/0.607) | Test | 2 | 95 | 83 | 957 | 100 | 0.487 | 0.534 | 0.487 | 0.92 | 0.509 |

*Abbreviations: FN, false negatives; FP, false positives; TN, true negatives; TP, true positives.*

# Table S3. SHAP-based feature importance for the telehealth reporting and telehealth intensity models

| **Feature** | **Reporting (P[Missing])** | **Intensity (log(1+VIDVZ) \| Reporters)** |
| --- | --- | --- |
| VTOT | 0.422 | 1.549 |
| MCRIPD | 0.327 | 0.281 |
| MCDIPD | 0.295 | 0.338 |
| SUROPTOT | 0.288 | 0.226 |
| Exp_per_Bed_M | 0.237 | 0.603 |
| Surg_Intensity | 0.22 | 0.23 |
| EXPTOT | 0.131 | 0.392 |
| ADMTOT | 0.122 | 0.285 |
| PAYTOT | 0.118 | 0.347 |
| CBSATYPE_NUM=2 | 0.057 | 0.053 |
| MAPP3=1 | 0.031 | 0.095 |
| CBSATYPE_NUM=0 | 0.024 | 0.041 |
| MAPP5=1 | 0.016 | 0.058 |
| MAPP3=2 | 0.009 | 0.022 |
| MAPP5=2 | 0.006 | 0.018 |
| MAPP8=1 | 0.005 | 0.016 |
| CBSATYPE_NUM=1 | 0.003 | 0.026 |
| MAPP8=2 | 0.001 | 0.0 |

*Variable definitions are provided in eTable 1.*

*Reporting model values are mean absolute SHAP values for predicting telehealth nonreporting among all hospitals (N = 6173).*

*Intensity model values are mean absolute SHAP values for predicting log-transformed telehealth intensity among reporting hospitals (N = 2652).*

# Table S4. Predictive performance of the telehealth reporting and telehealth intensity models

| **Model** | **Metric** | **Value** |
| --- | --- | --- |
| Reporting | Test AUC | 0.814 |
| Reporting | Test Accuracy | 0.728 |
| Reporting | N (all hospitals) | 6173 |
| Intensity | Test R2 | 0.41 |
| Intensity | Test RMSE | 3.213 |
| Intensity | Test MAE | 2.431 |
| Intensity | N (reporters) | 2652 |

*Reporting model performance was evaluated on the held-out test set among all hospitals.*

*Intensity model performance was evaluated on the held-out test set among hospitals that reported telehealth volume.*

# Table S5. Domain-grouped SHAP importance in the competing-driver models

| **Domain** | **Clinical AI** | **Operational AI** | **Total** |
| --- | --- | --- | --- |
| Mission: Teaching | 0.026 | 0.029 | 0.054 |
| Scale: Bed size | 0.159 | 0.193 | 0.352 |
| Complexity: Surgery | 0.207 | 0.213 | 0.42 |
| Finances: Margin | 0.287 | 0.32 | 0.607 |
| Demo: Medicaid % | 0.398 | 0.402 | 0.8 |
| Ops: Occupancy | 0.47 | 0.354 | 0.824 |
| Telehealth measures | 1.124 | 0.837 | 1.961 |

*Features were grouped into conceptual domains, including mission, scale, complexity, finances, demographics, operations, and telehealth measures.*

*Values represent aggregated SHAP importance for predicting high adoption in the clinical AI and operational AI models.*

# Table S6. Ablation analysis: change in test-set discrimination after removing telehealth

| **Target** | **Test AUC, full model** | **Test AUC, without telehealth** | **Relative drop, %** | **95% CI, low** | **95% CI, high** |
| --- | --- | --- | --- | --- | --- |
| Clinical AI (Tier 2) | 0.894 | 0.865 | 3.198 | 1.289 | 4.972 |
| Operational AI (Tier 2) | 0.85 | 0.839 | 1.302 | -0.64 | 3.004 |

*High adoption was defined as tier 2. Reported values compare the full competing-driver models with models in which telehealth was removed.*

# Table S7. Sensitivity analyses using reporter-only samples and continuous AI scores

| **Analysis** | **Domain** | **Sample N** | **Outcome** | **Performance** | **Key telehealth result** |
| --- | --- | --- | --- | --- | --- |
| Reporter-only tier model | Clinical | 2652 | Clinical AI Tier | Accuracy = 0.653; κ = 0.374; Tier 2 AUC = 0.821 | log telehealth volume ranked 1st; SHAP = 0.274 |
| Reporter-only tier model | Operational | 2652 | Operational AI Tier | Accuracy = 0.633; κ = 0.329; Tier 2 AUC = 0.775 | log telehealth volume ranked 1st; SHAP = 0.198 |
| Continuous-score model | Clinical | 6173 | Clinical AI Score | R² = 0.590; RMSE = 6.085; MAE = 3.851 | nonreporting ranked 1st; log volume ranked 2nd |
| Continuous-score model | Operational | 6173 | Operational AI Score | R² = 0.545; RMSE = 4.363; MAE = 2.763 | nonreporting ranked 1st; log volume ranked 2nd |
